# Antioxidant potential, *in vitro* cytotoxicity and apoptotic effect induced by crude organic extract of * Anthracophyllum lateritium against RD sarcoma cells*

**DOI:** 10.1186/s12906-015-0924-9

**Published:** 2015-11-06

**Authors:** Dilusha M Fernando, Ravi LC Wijesundera, Preethi Soysa, Dilip de Silva, Chandrika M Nanayakkara

**Affiliations:** Department of Plant Sciences, Faculty of Science, University of Colombo, Colombo 03, Sri Lanka; Department of Molecular Biology and Biochemistry, Faculty of Medicine, University of Colombo, Colombo 03, Sri Lanka; Department of Chemistry, Faculty of Science, University of Colombo, Colombo 03, Sri Lanka

**Keywords:** Macrofungi, Traditional medicine, *Anthracophyllum lateritium*, Antioxidant activity, Cytotoxicity, Apoptosis

## Abstract

**Background:**

Macrofungi have an established history of use in traditional oriental medicine. *Anthracophyllum lateritium* is a terrestrial macrofungus found in the dry zone forest reserves in Sri Lanka. Yet there are no scientific reports on bioactive properties of this species. Hence, the current study was aimed at determining the antioxidant potential, *in vitro* antiproliferative activity and apoptotic effect induced by crude methanolic extract of *A. lateritium* against RD sarcoma cell line.

**Method:**

The crude extract of *A. lateritium* was dissolved in methanol (MEFCA) and antioxidant activity was evaluated using *in vitro* assays: inhibition of DPPH (1,1-diphenyl-2-picrylhydrazyl) radical scavenging, ferric ion reducing power and 2-deoxy-D-ribose degradation assay. Total phenol and flavonoid contents of MEFCA were assayed using folin Ciocalteu method and aluminium chloride colorimetric method. *In vitro* cytotoxicity was determined using MTT assay against RD cells after 24 h exposure to MEFCA. Ethidium bromide/ acridine orange staining, DNA fragmentation and protein synthesis experiments were used to study the apoptotic features and antiproliferative activities of the treated cells. Glutathione assay and griess nitrite assay were used to analyze the reduced glutathione content and liberation of nitric oxide from apoptotic cells.

**Results:**

MEFCA showed promising antioxidant activity with EC_50_ values of 8.00 ± 0.35 μg/mL for DPPH scavenging and 83.33 ± 0.45 μg/mL for 2-deoxy-D-ribose degradation assay. The phenolic content was 265.15 ± 0.46 of (w/w) % of Gallic acid equivalents and flavonoid content was 173.01 ± 0.35 of (w/w) % of Epigallocatechingallate. *A. lateritium* showed strong *in vitro *cytotoxic activity with an EC_50_ of 18.80 ± 4.83 μg/mL for MTT assay against RD cells. Ethidium bromide/acridine orange staining and DNA fragmentation indicated the apoptotic features of treated cells. Protein levels showed a dose dependent decrease supporting the fact that *A. lateritium* induces apoptosis of treated cells. Glutathione content and nitric oxide content of cells exhibited a dose dependent increase suggesting the apoptosis of RD cells was mediated by both nitrie ions and nitric oxide.

**Conclusions:**

The crude extract of the *A. lateritium* exhibited potent antioxidant, antiproliferative activity and apoptotic effect against RD cells providing supportive evidence for the ethnopharmacological use of this fungus in control of oxidative damage and remedy of cancer.

## Background

Attributing to deep and firm roots in uses of macro fungi in the traditional medical practice of the far Eastern and recently in western world, constituent molecules of macro fungi organelles and secondary metabolites have long been believed to have important pharmacological properties such as antioxidant, antimicrobial, anticancer, cholesterol lowering and immunostimulatory effects [1, 2]. The active metabolites of the medicinal mushrooms such as *Ganoderma lucidum*, *Ramaria flava, Russula delica, Thelephora ganbajan* are found to have health promoting properties including antioxidant and antitumor activity [3]. Numerous publications indicate that the most important hazard for human diseases is posed by uncontrolled production of reactive oxygen species (ROS), including free radicals [4]. All living cells, including human cells, are continuously exposed to a variety of stress conditions leading to generate reactive oxygen species. From all free radical species, ˙OH and ˙O_2_- radicals are mainly involved in the oxidative damage, induces in the biological systems [5]. Natural antioxidants in the body act as a major defense against radical mediated toxicity by protecting the damage caused by free radicals [6, 7]. However, the endogenous mechanisms involved in free radical scavenging in the living cell sometimes become unbalanced and may be inadequate to neutralize the free radicals generated excessively. Hence, abundance of free radicals leads to be the major cause for deleterious conditions such as cancer and other degenerative diseases including cardiovascular diseases and hypercholesterolemia [8–10]. In spite of the availability of novel antineoplastic agents, cancer remains as the second leading cause of death affecting millions of people per year. The recent cancer therapies such as radiotherapy, chemotherapy and hormonal therapy has been made a modest progress in reducing the morbidity and mortality caused by cancer to the expected level [11, 12]. On contrary, molecules derived from natural sources including fungi and plants continue to play a dominant role in the discovery and development of novel and effective drug leads for cancer with minimal side effects. Currently, the medicinal importance of the wild mushrooms are being studied widely for their capacity to protect living cells and organisms from cancer [13, 14]. Being a tropical country, Sri Lankan biota has enormous fungal diversity and consists of a variety of macrofungi species with medicinal and aromatic values. Although, some of the species are used in traditional medicine, most of them are still not explored scientifically for their medicinal values [15]. *Anthracophyllum lateritium* is a terrestrial basidiomycete which is rarely found in the dry zone forest reserves of Sri Lanka. It belongs to the family of marasmiaceae. *A. lateritium* is a small shelf like fungus grown in large numbers. There is no stalk and caps are smooth with dull brown color [16]. Although, there are no reported studies on biological activities of *A. lateritium*, *Lentinus edodes* which belongs to the same family (marasmiaceae) has shown important bioactive properties including antioxidant and antiproliferative activity [16]. Hence, the current study is an extended effort to broaden and uncover the antioxidant and cytotoxic properties of *A. lateritium* which leads to development of drug leads in the treatment of cancer and other degenerative diseases. The fruiting bodies of the macrofungus were used to determine the biological activities.

## Methods

### Chemicals and equipments

Folin ciocalteu reagent, sodium carbonate (Na_2_CO_3_), aluminium chloride (AlCl_3_), sodium nitrite (NaNO_2_), sodium hydroxide (NaOH) and chemicals needed for cell culture and cytotoxicity studies were purchased from Sigma–Aldrich (P.O. box 14 508, St Louis, MO 63178 USA). 1-Diphenyl-2-picrylhydrazyl (DPPH), Triton X-100 solution (1 %), gallic acid, sulfanilamide and ortho-phosphoric acid, (−)-Epigallocatechinegallate were purchased from Fluka (Fluka chemie GmbH, CH – 9471 Buchs). Ascorbic acid, methanol and Dichloromethane were purchased from BDH Chemicals (Poole, England). Tris base was purchased from Promega (Promega Corporation, Madison, WI 53711–5399, USA). All chemicals used were of analytical grade.

Shimadzu UV 1601 UV visible spectrophotometer (Shimadzu Corporation, Kyoto, Japan) was used to measure the absorbance. Rotatory evaporator (BUCHI Rota vapor R-200) was used to obtain the crude extract of *A. lateritium*. Cells were incubated at 37 °C in a humidified CO_2_ incubator (SHEL LAB/Sheldon manufacturing Inc. Cornelius, OR 97113, USA). Olympus (1X70-S1F2) inverted fluorescence microscope (Olympus Optical Co. Ltd. Japan) for observation of cells and photographs were taken using a Nikon D700 camera (Nikon D700, Japan). Deionized water from LABCONCO (waterproplus) UV ultra-filtered water system (LABCONCO Corporation, Kansas city, Missouri 64132–2696) or distilled water was used in all experiments.

### Fungal material

The specimen of *A. lateritium* was collected from the dry zone forest reserves of Dambulla in Sri Lanka during the period of September 2012 to October 2013. They were collected into paper bags and packed loosely with proper ventilation during the transportation. The identity of the specimen was achieved by the Department of Plant Science, Faculty of Science, University of Colombo (Genbank Accession No.: KP757737). Voucher specimens were deposited at the same institute (UOC:DAMIA:D26).

### Preparation of the extract

Mature fruiting bodies of *A. lateritium* were brush cleaned, dried in the oven at 40 °C to a constant mass and pulverized. The dried powder of each specimen (10 g) was sonicated sequentially with 150 mL of 100 % methanol, 99 % dichloromethane and methanol: dichloromethane (1:1) mixture at 30 °C for 1 h. Each extract was filtered twice through Whatman No. 1 filter paper. The filtrates were pooled together and evaporated to dryness at 40 °C under reduced pressure using rotatory evaporator to obtain the crude extract. Crude extract was dissolved in methanol and used for further experiments (MEFCA).

### Determination of antioxidant activity

Antioxidant activity by 1, 1- diphenyl-2-picrylhydrazyl (DPPH) radical, ferric ion reducing power and nonsite-specific hydroxyl radical mediated 2- deoxy-D-ribose degradation of the MEFCA were determined according to the methods described previously with modifications [17]. The percentage antioxidant capacity (% AI) was calculated from the following equation: % AI = [(absorbance of control-absorbance of sample)/absorbance of control] x 100 %. The effective concentration of the sample required to scavenge the respective radical by 50 % (EC_50_) was calculated using the linear segment of the curve obtained with % AI against concentration.

### Total phenol content (TPC) and total flavonoid content (TFC)

TPC and TFC of the MEFCA were determined by the Folin - Ciocalteau method [18, 19] and slightly modified method of aluminium chloride colorimetric assay [20].

### Cell lines and cell culture

The human muscle rhabdomyosarcoma (RD) cell line was used to assess the cytotoxicity of the MEFCA. The RD cell line is derived from a biopsy of 7 year old child having pelvic refractory RMS, previously treated with cyclophosphamide and radiation (McAlliste et al., 1969). RD cell line was obtained from the Medical Research Institute, Colombo 08, Sri Lanka. The cells were cultured in DMEM supplemented with 10 % heat inactivated fetal bovine serum (FBS), HEPES, 3 % glutamine, sodium bicarbonate and antibiotic (penicillin/streptomycin) and incubated at 37 °C in a humidified CO_2_ incubator.

### MTT assay

Cytotoxic activity was determined using MTT cell viability assay after 24 h treatment of the MEFCA to RD cells. Metabolically active cells reduce MTT (3, 4, 5-(dimethyl-thiazol-2-yl) 2-5-diphenyl tetrazolium bromide) in to purple colored formazan crystals. RD cells (2 × 10 ^5^ cells/well) were seeded in 24-well plates and incubated overnight with 1 mL of the medium described above. The resulting monolayer of cells (70 % confluence) was treated with different concentrations of the MEFCA (1–150 μg/mL) and incubated for 24 h at 37 °C. In all experiments, cycloheximide (5 mM, 50 μL) was used as the positive control and negative control contained only the growth media. The culture medium was replaced with fresh medium (1 mL) and MTT (5 mg/ml; 100 μL) was added to each well. The cells were incubated at 37 °C for 3 h and the medium was aspirated carefully. The remaining formazan crystals were solubilized with 750 μL of 0.05 M HCl (in 2-propanol) and absorbance was measured at 570 nm. Percentage cell viability = [(Absorbance of untreated cells -Absorbance of treated cells)/Absorbance of untreated cells]*100. The net absorbance from the wells of the untreated cells (negative control) was taken as the 100 % viability. EC_50_ was determined by regression analysis of the corresponding dose response curve.

### Morphological determination

The morphological changes of RD cells after the treatment with different concentrations of the MEFCA over 24 h were detected by microscopic examination of cells. Morphological changes were compared with negative and positive controls under fluorescence microscope.

### Ethidium bromide/Acridine orange staining

Ethidium bromide and acridine orange staining was performed to investigate the apoptosis induced by MEFCA against RD cells. Ethidium bromide (EB) is only occupied by cells with damaged cell membranes. Acridine orange (AO) permeates via both live and dead cells, while stains the DNA making nucleus appear in green. Thus, live cells will be uniformly stained green. Apoptotic cells will be stained in orange or red with acriding orange depending on the degree of loss of membrane integrity and co-staining with ethidium bromide. Cells were seeded in chamber slides and the confluent layer was treated with the MEFCA at different concentrations for 24 h at 37 °C as described previously. The adherent cells were washed with 200 μL of PBS and 2 μL of the dye mixture containing ethidium bromide (100 mg/mL) and acridine orange (100 mg/mL) was placed on each well of the chamber slide. Chamber slides were examined immediately under the florescence microscope. Images were photographed using a Nikon D 700 camera connected to microscope.

### DNA fragmentation assay

RD cells were seeded in culture flasks (25 mL) and the confluent layer was treated with the MEFCA at different concentrations for 24 h at 37 °C as described previously. The DNA fragmentation assay was carried out according to a previously described method [21].

### Estimation of protein content

RD cells were seeded in 24 well plates and the confluent layer was treated with the MEFCA at different concentrations (2–60 μg/mL) for 24 h at 37 °C as described previously. The cell lysates were obtained by dissolving the treated monolayers of cells in 1 mL of 0.1 % Triton-X solution. The protein content in the cell lysate was determined using a method described previously [22].

### Griess nitrite assay

The nitric oxide content of the supernatant obtained from RD cells seeded in 24 well plates treated with MEFCA was estimated using the griess nitrite assay [23]. The nitrite content was calculated based on a NaNO_2_ standard curve.

### Determination of reduced glutathione

The reduced glutathione (GSH) content of the RD cell lysates was determined using the methods described previously with slight modifications [24, 25]. A series of reduced glutathione standards (0.5 – 3 μg/mL) treated in a similar manner were also performed to determine the reduced glutathione content in cell lysate.

### Calculations and statistics

All experiments were performed in triplicate and values shown are representative for at least three independent experiments. All the results of the experiments were expressed as the mean ± standard deviation (Mean ± SD). Least square linear regression analysis was applied using Microsoft excel and linear segment of the curve of percentage inhibition plotted with concentration was used to determine the EC_50_ values. R^2^ > 0.99 was considered as linear for the calibration curves.

## Result

### Antioxidant activity

**Fig. 1:**
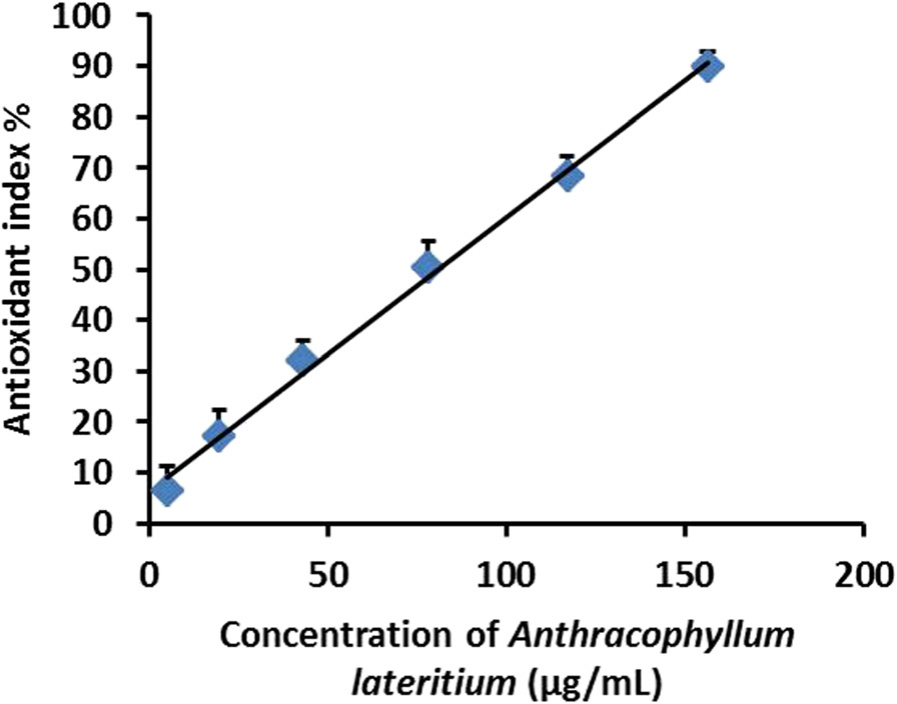
Dose response curves for DPPH radical scavenging capacity of MEFCA. The % antioxidant index was plotted against concentration of the sample. The graphical data are represented as mean ± SD of three independent experiments

**Fig. 2:**
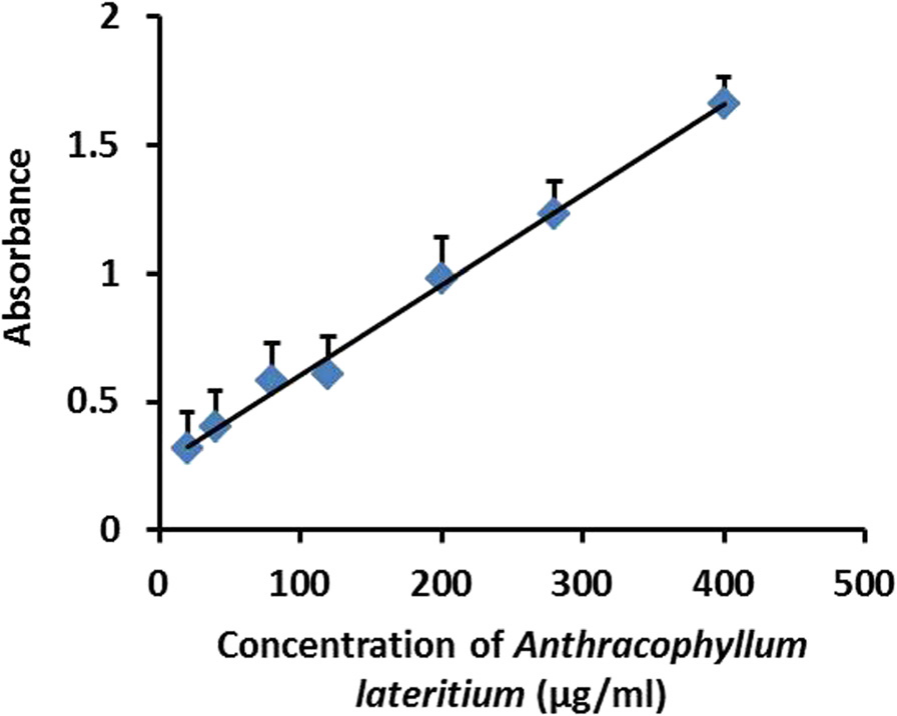
Ferric ion reducing power assay for MEFCA. The absorbance (A_700_) was plotted against concentration of the sample. The graphical data are represented as mean ± SD of three independent experiments

**Fig. 3:**
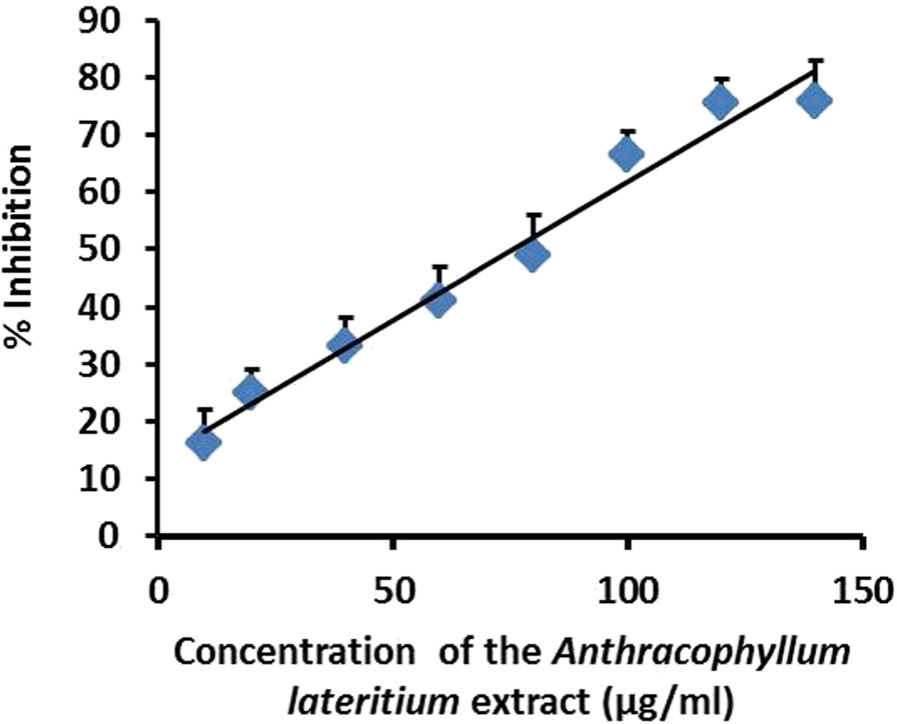
Hydroxyl radical mediated 2-deoxy-D-ribose degradation assay for MEFCA. The % inhibition was plotted against concentration of the sample. The graphical data are represented as mean ± SD of three independent experiments

The EC_50_ value which is the effective concentration of the MEFCA required to scavenge DPPH radicals by 50 % was 8.00 ± 0.35 μg/mL (Fig. 1). L- Ascorbic acid was used as the standard antioxidant to compare the EC_50_ value obtained for MEFCA. Interestingly, EC_50_ calculated for MEFCA was closer to the EC_50_ value obtained for ascorbic acid (5.50 ± 0.45 μg/mL) implying its strong radical scavenging activity. In Ferric ion reducing power assay, MEFCA showed strong reducing power forming blue or green coloured complex with reduction of Fe^3+^/ferricyanide complex in to the ferrous form depending on the reducing power of each concentration of the extract. Ferric ion reducing power was gradually increased in a dose dependent manner in the range of 20–400 μg/mL (Fig. 2). The effective concentration of the MEFCA required to scavenge the reactive hydroxyl radicals by 50 % (EC_50_) evaluated by hydroxyl radical mediated 2- deoxy-D-ribose degradation was 83.33 ± 0.45 μg/mL (Fig. 3). The value was higher than the standard antioxidant, gallic acid (10.04 ± 0.27 μg/mL).

### Phenolic and flavonoid content

The phenolic content of the MEFCA was 265.15 ± 0.46 of Gallic acid equivalents (GAE)/dry weight (mg) of sample and flavonoid content was 173.01 ± 0.35 of Epigallocatechingallate (EGCG)/dry weight (mg) of sample.

### Cytotoxicity and apoptosis assays

#### MTT assay

**Fig. 4:**
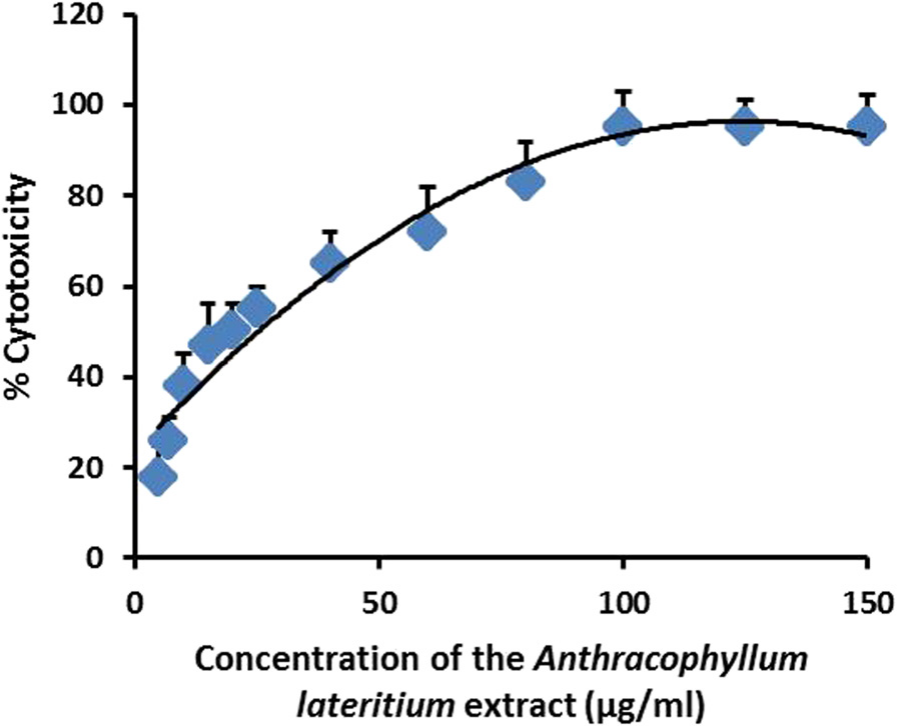
The percentage induction of cytotoxicity (inhibition of cell proliferation) against RD cell line as determined by MTT assay, after 24 h treatment with the MEFCA. The graphical data are represented as mean ± SD of three independent experiments

A dose response curve of percentage cell viability against the concentration of the extract was obtained using result obtained for MTT cell viability assay (Fig. 4). The EC_50_ value obtained for the mean of the three independent sample preparations against RD cells was 18.80 ± 4.83 μg/mL. Positive control (cycloheximide) exhibited 78.45 ± 2.22 % growth inhibition at the concentration (5 mM, 50 μL) used.

### Morphological changes

**Fig. 5:**
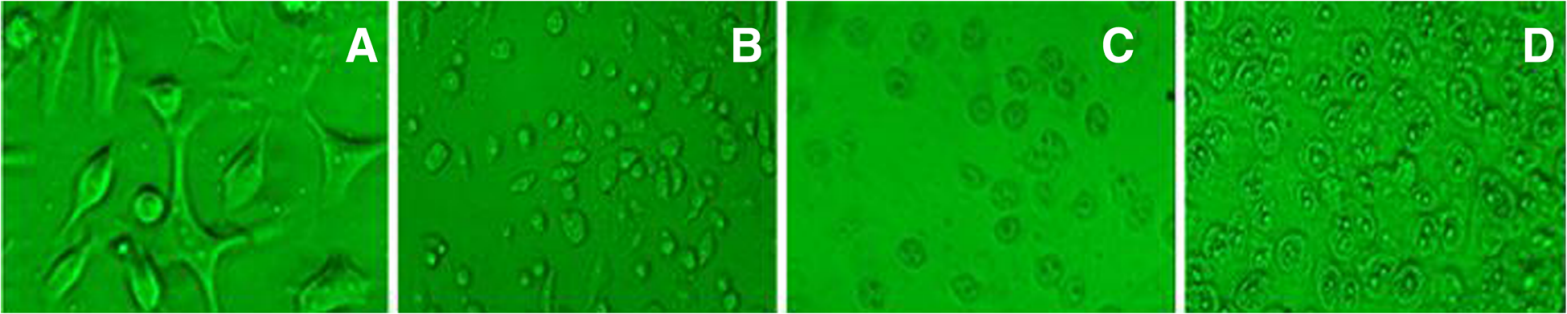
Fluorescent micrographs of RD cell line after 24 h of incubation with the MEFCA at different concentrations. **a**-negative Control; **b**-Positive Control (Cycloheximide); **c**- 20 μg/mL; **d**- 100 μg/mL. Live cells have characteristic polygonal shape and dead cell are rounded. Reduction in cell density was also observed in positive control and the treated cells with highest concentration (Original magnification 20×)

The morphological changes observed on RD cells after treatment of MEFCA are represented in Fig. 2. The cell morphology of untreated cells appeared in elongated shape and treated cells appeared with cellular shrinkage, oval or irregular in shape and condensed cytoplasm. The cells treated with highest concentration of the MEFCA (100 μg/mL) displayed the detached and round shape dead cells as indicated in Fig. 5.

### Ethidium bromide/acridine orange staining

**Fig. 6:**
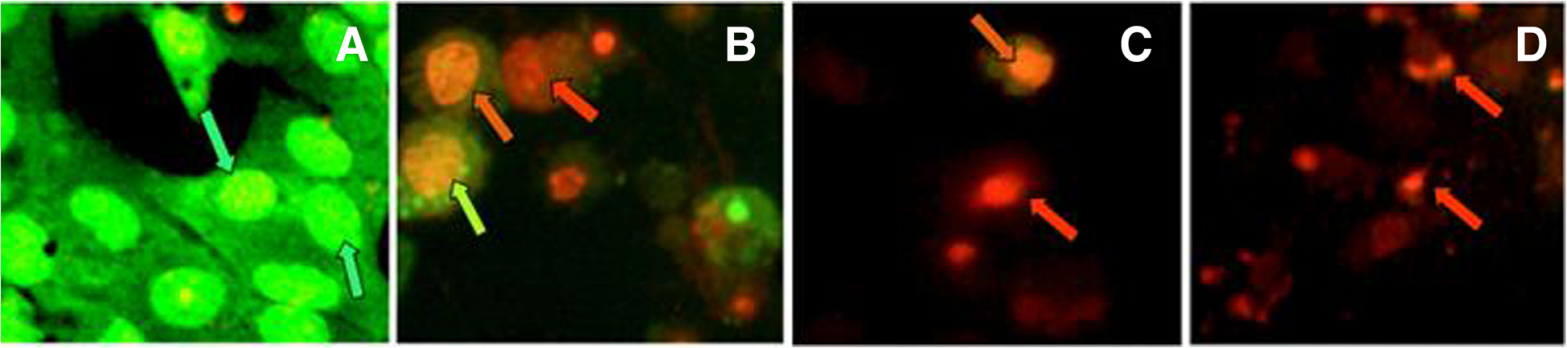
Apoptotic morphology detection by Acridine orange-ethidium bromide (AO/EB) fluorescent staining of RD cell line treated with the MEFCA. **a**-negative control; **b**- cycloheximide as the positive control (5 mM; 50 μL); **c**- 20 μg/mL, **d**- 50 μg/mL of the extract. Green arrows: live cells, greenish yellow: early apoptotic cells, orange red: late apoptotic cells, red: dead cells (some cells are fragmented and become faded). This figure denotes the results of at least 3 independent experiments (Original magnification 40×)

Morphological changes of cells stained with ethidium bromide/acridine orange (1:1) after treatment of extract indicated that untreated cells (negative control) appeared with the normal nuclei presented bright green. Early apoptotic cells showed greenish yellow nuclei and late apoptotic cells indicated condensed orange red nuclei. Dead cells appeared in red. Chromatin condensation, nuclear fragmentation, presence of apoptotic bodies and blebbing formation of apoptotic cells were also evident upon examination of stained cells with ethidium bromide/acridine orange (Fig. 6).

### DNA fragmentation

**Fig. 7:**
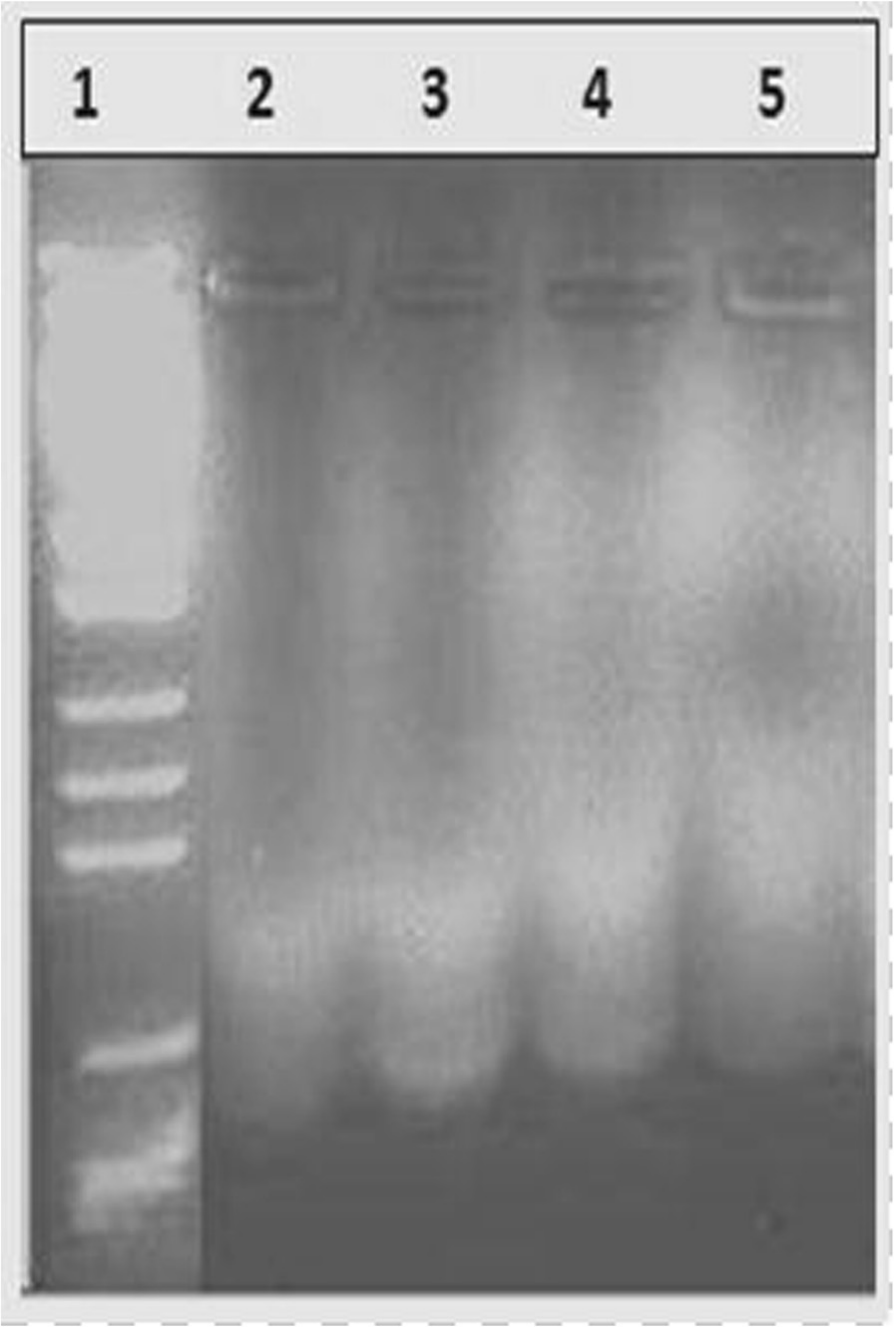
Induction of apoptosis by DNA fragmentation shown by Agarose gel electrophoresis. Lane 1: DNA molecular weight marker, Lane 2: 10 μg/mL, Lane 3: 20 μg/mL of extract, lane 4: 50 μg/mL of extract, Lane 5: 100 μg/mL

DNA fragmentation was observed at a concentration of 10, 20, 50, 100 μg/mL of MEFCA after 24 h exposure (Fig. 7). A unique ladder pattern and DNA shearing related to apoptosis was observed by DNA extracted from treated cells with fungal extract. The higher dose (100 μg/mL) of the extract induced significantly greater fragmentation or shearing of the DNA compared to lower concentrations.

### Estimation of protein content

**Fig. 8:**
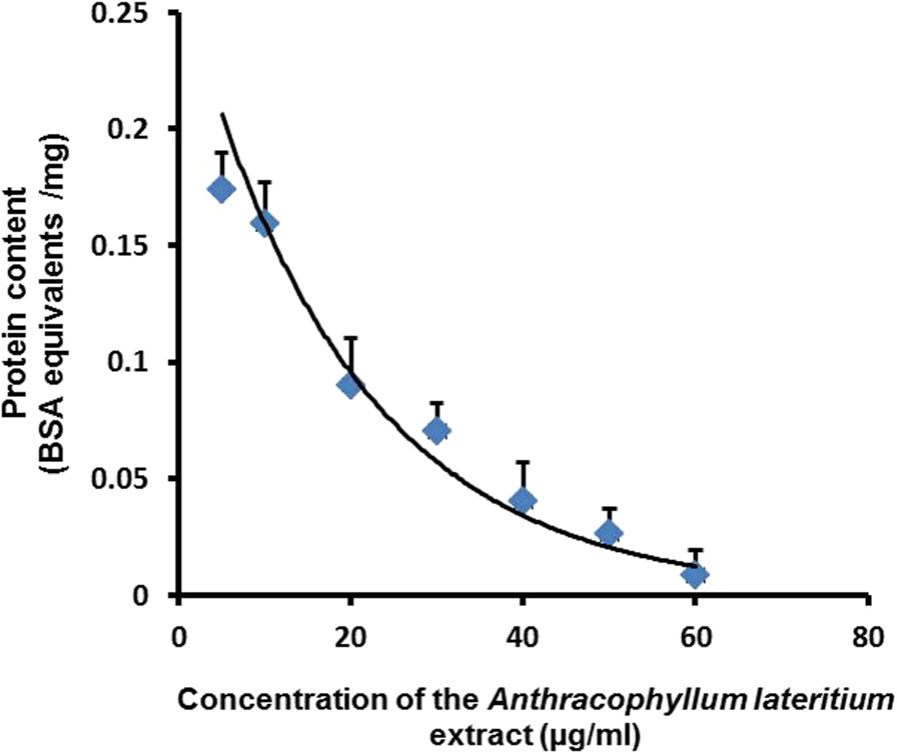
Protein content in the RD cell lysate after 24 h treatment with the MEFCA. The graphical data are represented as mean ± SD of three independent experiments

The protein levels of the cell lysates of treated RD cells exhibited a dose dependent decrease as shown in Fig. 8.

### Griess nitrite assay

**Fig. 9:**
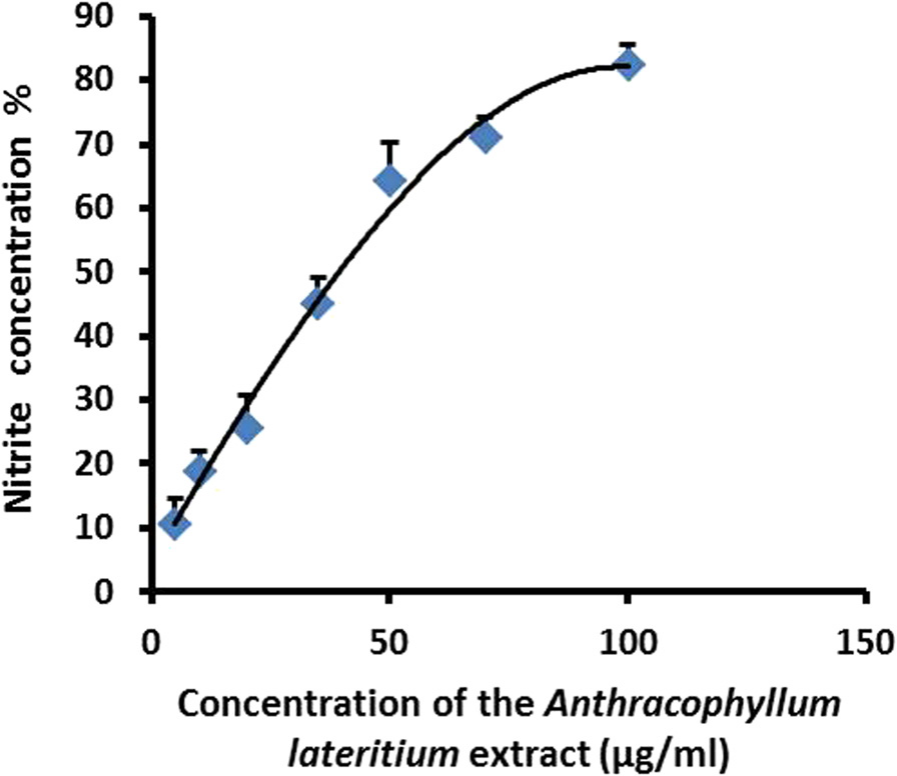
Nitrite content in the RD cell supernatant after 24 h treatment with the MEFCA. The graphical data are represented as mean ± SD of three independent experiments

Nitric oxide (NO) content in the cell supernatant analyzed by griess nitrite assay showed a dose dependent increase compared to the negative control (Fig. 9).

### Determination of reduced glutathione

**Fig. 10:**
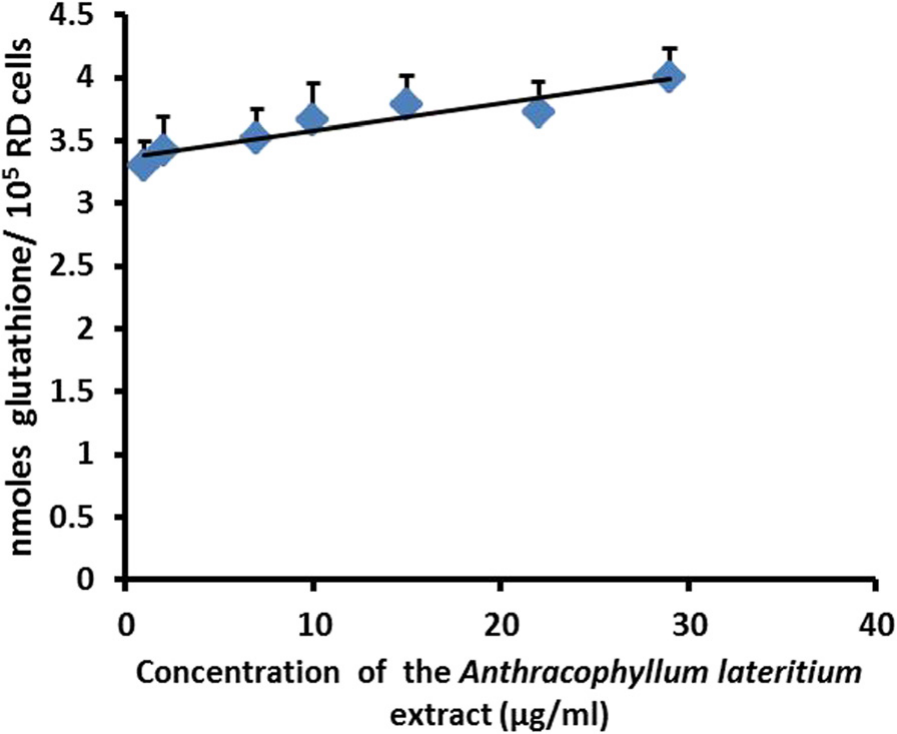
The reduced glutathione (GSH) content in the RD cell lysate after 24 h treatment with the MEFCA. The graphical data are represented as mean ± SD of three independent experiments

The reduced glutathione (GSH) content in the RD cell lysate showed a dose dependent increase compared to the negative control (Fig. 10).

## Discussion

Among the secondary metabolites produced by macrofungi, bioactive phenols and flavonoid derivatives are of predominant due to their strong capacity for free radical scavenging. Mainly, polyphenols have gained major importance in scavenging free radicals which is correlated to their chemical structure consisting of an aromatic ring with hydroxyl substituents [26]. This effect is directly involved with subsidizing anticancer activity against human cancer cells. Therefore, there is a recent upsurge in the interest of mushrooms, due to their high content of polyphenol, which positively correlates with the antioxidant activity and antiproliferative effect [27–29].

In the current study, MEFCA showed promising antioxidant activity with DPPH, Ferric ion reducing power, and OH radical scavenging capacities. DPPH scavenging capacity was found to be highest, followed by hydroxyl radical scavenging activity. Also it exhibited in vitro cytotoxicity and large quantities of phenol and flavonoid compounds. These findings suggest high polyphenol content of fungi largely contribute for strong antioxidant and antiproliferative activity. Compelling cytotoxic activity against RD cell line was found using MTT cell viability assay which is a well-established method to assess the cell viability. As evident in Fig. 1, dose dependent increase was observed for *in vitro *cytotoxicity for RD cell line over a range of 2–80 μg/mL of MEFCA. Interestingly, a maximum of 95 % inhibition of cell growth was observed at concentrations over 80 μg/mL. Antiproliferative activity of MEFCA was found to be approximately eight times greater than the activity shown by *Ganoderma lucidum* which is an important medicinal mushroom used today, acclaimed as “mushroom of immortality”. *G. lucidum* possesses an anticancer effect with an EC_50_ of 125.00 ± 0.03 μg/mL as evaluated by MTT assay [30, 31]. In addition, methanol extract of *G. lucidum* exhibited antioxidant activity on the 1,1-diphenyl-2-picrylhydrazyl radical with an EC_50_ value of 1.162 ± 0.016 mg/mL [32]. This is found to be seventy times lower than DPPH antioxidant activity shown by MEFCA. *Pleurotus cystidiosus* is another important edible mushroom which possesses antiproliferative activity. It has been shown cytotoxic properties against Hep-2 cells with an EC_50_ value of 3.6 mg/mL for a fraction of methanol:water (7:3) [33]. MEFCA also exhibits strong antiproliferative activity compared to the activity shown by *P. cystidiosus.*

Distinct morphological changes observed on tumor cells after treatment of extract of *A. lateritium,* compared to negative control reveal its antitumor activity. Microscopic examination of cell morphology of treated cells following 24 h incubation exhibited characteristic features of dead cells which were then further analyzed by ethidium bromide/acridine orange staining method. Cells stained with AO/EB in yellowish green indicate the signs of early apoptosis and orange red represents late apoptotic cells. Chromatin condensation and nuclear fragmentation were predominantly observed apoptotic features for treated cells with high concentrations of the extract and quantity of apoptotic cells was gradually increased with the treatment dose. Morphological characterization of treated cells revealed that the mode of action of cell death induced by the methanol extract of *A. lateritium* was mediated through apoptosis. It strengthens the results obtained by MTT assay.

This was further confirmed by the DNA fragmentation assay indicating a unique ladder banding pattern (Fig. 4). Cleavage of oligonucleosomal DNA at the internucleosomal linker sites yielding DNA fragments in multiples of 180 bp is reflected as the biochemical hallmark of apoptosis [34, 35]. Appearance of such fragments resulting in a ladder formation was evident when fragmented DNA was isolated from apoptotic cells treated with the respective drug/extract or decoction and subjected to agarose gel electrophoresis.

Protein stability is a key regulatory mechanism in the control of cell growth, cell cycle and apoptosis. The selective degradation or stabilization of intracellular proteins by ubiquitin-dependent pathways is essential for the regulation of many cellular processes. During apoptosis, regulatory and other proteins have been identified as target substrates for ubiquitination [36]. Apoptosis is massively occurred in treated cells with high doses of MEFCA. Hence, protein levels are also gradually decreased with the treatment dose of the extract. The result reveals that protein levels of the treated cells exhibited a dose dependent decrease.

Nitrite content in the cell supernatant investigated by the griess nitrite assay showed a dose dependent increase implying the role of NO in tumor regression. The role of NO in mediating tumor regression has been reported in a number of studies [37–39]. Higher NO levels are known to suppress metastasis via alterations of the expression of apoptosis associated proteins. Nitric oxide (NO) is synthesized endogenously by a family of NO synthases and has been demonstrated to involve in a variety of biological signaling processes, including apoptosis. As NO is a highly reactive free radical within biological systems, it can react with other free radicals, molecular oxygen and heavy metals. NO can react rapidly in the intracellular environment to form nitrites, which in turn analyzed by griess nitrie assay. It has been reported that the biological effects of NO can be also mediated by the products of different NO metabolites. These metabolites including nitrites are known to play a vital role in mediating genotoxic effects via DNA damage caused by DNA modification or DNA strand breakage. This NO metabolites mediated DNA damage induces cellular apoptosis [40–42]. Therefore, it is evident that generated nitrite ions from NO played a significant role in regression of RD cell growth. These findings suggest the apoptosis of RD cells was mediated by both nitrie ions and nitric oxide.

NO increases TCA cycle flux via increase in glucose uptake and increase in cellular glutamine. This potential link between the increase in TCA cycle activity and NO in cancer cells may result in the production of glutathione (GSH) in tumor cells [43]. In this study, the reduced glutathione (GSH) content in the cell lysate showed a dose dependent increase against RD cell line supporting the positive correlation between nitric oxide content and production of glutathione in tumor cells. It augments the result obtained from griess nitrite assay.

Importantly, the crude organic extract of *A. lateritium* possesses potent anticancer and antioxidant effect revealing its pharmacological importance. To the best of our knowledge, this is the first report on antioxidant activity and *in vitro* cytotoxicity of *A. lateritium*. This appears to be a potential source of novel anticancer and antioxidant compounds.

## Conclusions

The methanol extract of *A. lateritium* demonstrated high antioxidant potential to variable levels as analyzed by different antioxidant assays. It suggests that *A. lateritium* is a potential therapeutic agent for the control of oxidative damage caused by free radicals. The potent anticancer and apoptotic effect induced by crude extract of *A. lateritium* observed in the present study imply the ethnopharmacological potential of this fungus in remedy of cancer. It can be emerged as an important source of antioxidant and antitumor compounds. Further, present findings suggest there might be a direct involvement of phenolic and flavonoid compounds to the observed promising anticancer and antioxidant effect of *A. lateritium.* This study provides a scientific proof of the traditional awareness in using medicinal mushrooms as an anticancer agent.

## Abbreviations

AO/EB: Acridine orange (AO)/ethidium bromide (EB)
DPPH: 1-Diphenyl-2-picrylhydrazyl
MEFCA: Methanol extract of the crude organic extract of *A. lateritium*
MTT: 3 4, 5-(dimethylthiazol-2-yl) 2-5-diphenyl tetrazolium bromide
TPC/TFC: total phenol content/total flavonoid content
